# Comparison of the Effects of Powdered and Powder-free Surgical Gloves on Postlaparotomy Peritoneal Adhesions in Rats

**DOI:** 10.5812/ircmj.2272

**Published:** 2013-05-05

**Authors:** Arman Aghaee, Hossein Parsa, Marjan Nassiri Asl, Seyed Amir Farzam, Amir Javadi, Ashkan Divanbeigi

**Affiliations:** 1 Department of Surgery, Qazvin University of Medical Sciences, Qazvin, IR Iran; 2 Cellular and Molecular Research Centre, Department of Pharmacology, Qazvin University of Medical Sciences, Qazvin, IR Iran; 3 Department of Pathology, Qazvin University of Medical Sciences, Qazvin, IR Iran; 4 Department of Biostatistics, Qazvin University of Medical Sciences, Qazvin, IR Iran; 5 Shefa Neuroscience Research Center, Tehran University of Medical Sciences, Tehran, IR Iran

**Keywords:** Adhesion, Gloves, Surgical, Rat

## Dear Editors,

Most intra-abdominal adhesions result from peritoneal injuries caused by presurgical procedures or intra-abdominal infections. Adhesions were reported during postmortem examinations in 67% patients who had undergone presurgical procedures and in 28% patients with a history of intra-abdominal infections. The process of postoperative adhesion formation includes a complex interaction of biochemical events involved in inflammation, tissue repair, angiogenesis and innervations ( 1 ). Moreover, surgical trauma to the peritoneum can occur because of various mechanisms such as incision, abrasion, ischemia, desiccation and coagulation ( 2 ). General strategies such as surgical approach, surgical technique and surgical adjutants, including barriers and gels, are used for preventing postoperative adhesions ( 3 , 4). It has been suggested that peritoneal adhesions after surgery are common and remain a major problem for both the health and society. Therefore, we studied the effects of powdered and powder-free surgical gloves on postlaparotomy peritoneal adhesions in a rat model. Male Wistar rats (n = 60; weight, 250-300 g) were obtained from the Razi Institute (Karaj, Iran) and housed under standard laboratory conditions with free access to food and water. The rats were randomly divided into 2 groups comprising 30 rats each. In group 1, the procedure of laparotomy was performed using sterile powdered gloves (Sempermed, Austria). In group 2, the procedure was performed using sterile powder-free gloves (Supreme, Austria). Rats were anesthetised by intraperitoneal injection of ketamine (60 mg/kg) and xylazine (6 mg/kg). All animals underwent laparotomy, and 5 abrasions of 3–4 cm were made on the caecum and the peritoneal wall. Next, the caecum was placed back in the abdomen, and the abdomen was closed in 2 layers using a nylon string (2–0 inches) (Supa, Iran) and running sutures. After 14 days, all animals were anesthetised, and laparotomy was performed through the previous midline incision ( 5). Adhesions were graded by an observer blinded to the experimental groups. The adhesion grading scale was as follows: 0, absent; 1, thin and easily separable; 2, ﬁbrotic and requiring sharp dissection and 3, extensive and dense adhesions ( 6 ). Biopsy samples were collected from the caecum with postoperative adhesions. These samples were then prepared for histopathological examination and observed under a microscope. Statistical analysis was performed using the chi-square and t-tests. P < 0.05 was considered statistically significant. After the initial operation, none of the rats were found to have adhesions, and no complications or deaths were observed. As shown in Table 1, all types of adhesions were reported in the powdered gloves group. Most adhesions in this group were grade 3. A significant difference was observed between the 2 groups with respect to the adhesion grade 0 (P = 0.013). The percent adhesion score was higher in the powdered gloves group than in the powder-free gloves group. Results of histopathological examination also confirmed the grades of all the adhesions. 

**Table 1.** Number of Adhesions and Their Percent Scores in the 2 Groups on Postoperative Day 14

Types of adhesions	Powdered gloves, No. (%)	Powder-free gloves, No. (%)	Total
**1**	0 (0)	14 (46.7)^a^	14 (23.3)
**2**	8 (26.7)	4 (13.3)	12 (20)
**3**	8 (26.7)	8 (26.7)	16 (26.7)
**4**	14 (46.7)	4 (13.3)	18 (30)
**Total**	30 (100)	30 (100)	60 (100)

^a^P < 0.013

Several animal and human studies have analysed the role of starch-powdered gloves on the formation of adhesions (7). An injury to the peritoneum may be inflammatory or surgical and may be caused by exposure to infection or intestinal contents, ischemia and irritation from foreign materials such as sutures, gauze particles, glove-dusting powder, abrasion and desiccation (8). Detrimental effects of glove cornstarch on wound closure techniques have been established. Moreover, this powder potentiates wound infection. In addition, it serves as a carrier of latex allergens that cause life-threatening allergic reactions in sensitised patients (9). Our study reported that most adhesions were observed in the powdered gloves group. Thus, the use of powdered gloves during abdominal surgery needs to be justified.
